# Therapeutic targeting of the PI4K2A/PKR lysosome network is critical for misfolded protein clearance and survival in cancer cells

**DOI:** 10.1038/s41388-019-1010-4

**Published:** 2019-09-25

**Authors:** Apar Pataer, Bulent Ozpolat, RuPing Shao, Neil R. Cashman, Steven S. Plotkin, Charles E. Samuel, Steven H. Lin, Nashwa N. Kabil, Jing Wang, Mourad Majidi, Bingliang Fang, Jack A. Roth, Ara A. Vaporciyan, Ignacio I. Wistuba, Mien-Chie Hung, Stephen G. Swisher

**Affiliations:** 1 Departments of Thoracic and Cardiovascular Surgery, The University of Texas MD Anderson Cancer Center, Houston, TX USA; 2 Departments of Experimental Therapeutics, The University of Texas MD Anderson Cancer Center, Houston, TX USA; 3 Department of Medicine (Neurology), The University of British Columbia, Vancouver, BC Canada; 4 Vancouver Coastal Health Research Institute, Brain Research Centre, Vancouver, BC Canada; 5 Amorfix Life Sciences Ltd, Mississauga, ON Canada; 6 Department of Physics & Astronomy, The University of British Columbia, Vancouver, BC Canada; 7 Department of Molecular, Cellular and Developmental Biology, University of California, Santa Barbara, CA USA; 8 Departments of Radiation Oncology, The University of Texas MD Anderson Cancer Center, Houston, TX USA; 9 Departments of Bioinformatics, The University of Texas MD Anderson Cancer Center, Houston, TX USA; 10 Departments of Translational Molecular Pathology, The University of Texas MD Anderson Cancer Center, Houston, TX USA; 11 Molecular and Cellular Oncology, The University of Texas MD Anderson Cancer Center, Houston, TX USA

**Keywords:** Non-small-cell lung cancer, Target identification

## Abstract

The role of RNA-dependent protein kinase R (PKR) and its association with misfolded protein expression in cancer cells are unclear. Herein we report that PKR regulates misfolded protein clearance by preventing it release through exosomes and promoting lysosomal degradation of misfolded prion proteins in cancer cells. We demonstrated that PKR contributes to the lysosome function and regulates misfolded prion protein clearance. We hypothesized that PKR-associated lysosome function is critical for cancer but not normal cell survival, representing an effective approach for highly targeted cancer therapy. In screening a compound library, we identified two PKR-associated compounds 1 and 2 (Pac 1 and 2) did not affect normal cells but selectively induced cell death in cancer cells depending on their PKR expression status. Pac 1 significantly inhibited the growth of human lung and breast xenograft tumors in mice with no toxicity. Pac 1 binds to PI4K2A and disrupts the PKR/PI4K2A-associated lysosome complex, contributing to destabilization of cancer cell lysosomes and triggering cell death. We observed that PKR and PI4K2A play significant prognostic roles in breast cancer patients. These results demonstrate that targeting of a PI4K2A/PKR lysosome complex may be an effective approach for cancer therapy.

## Introduction

Researchers suggested that cancer cells depend on lysosome function for survival, as lysosomes eliminate abnormal proteins [1, 2]. Lysosomes break down misfolded proteins, an important defense mechanism against cell death triggered by aggregation of misfolded proteins in cells [3–5]. Proteins must fold into three-dimensional shapes to function properly. However, many factors can cause protein misfolding and create incorrect protein shapes (so-called misfolded proteins) [6, 7]. The most frequent destiny of misfolded proteins is self-aggregation [6]. Accumulated misfolded proteins may be assembled into toxic aggregates [6, 7]. Also, Accumulation of misfolded proteins can cause many diseases, including cancer, Alzheimer disease, Parkinson disease, Huntington disease, type 2 diabetes mellitus, inherited cataracts, atherosclerosis, hemodialysis-related disorders, and short-chain amyloidosis [6–8]. All humans are at risk for accumulation of misfolded proteins everyday, and to function properly, our cells must continually make proteins, with the understanding that misfolding will ultimately help protect us from serious diseases. Cancer cells may produce more misfolded proteins than normal cells do because of their high mutation rates, high metabolic demand, rapid growth, and aberrant glycosylation and the failure of host defense/clearance mechanisms [9–12]. In addition to “loss of function” due to alteration of the native state, a misfolded protein can exert pleotropic function and promote malignant growth and transformation through the activation of several of oncogenic pathways such as autophagy, unfolded protein response, and metabolic reprograming [9, 10, 12]. Therefore, a single misfolded protein can disturb function of many cellular proteins and alter multiple regulatory pathways. Increasing evidence demonstrates that high levels of expression of misfolded proteins or RNA-dependent protein kinase R (PKR) are associated with the development of neurodegenerative diseases, such as Alzheimer, Parkinson, and Huntington disease [13–17]. PKR has a well-established role in antiviral defense mechanisms and other cellular functions, such as growth control, apoptosis regulation, signal transduction, and differentiation [18–23]. However, the link of PKR expression with misfolded protein expression in cancer cells is unclear. Thus, further investigation of the role of PKR and its association with misfolded protein clearance in cancer is warranted.

In the present study, we demonstrated the new role of PKR on misfolded protein clearance in cancer cells. We found that PKR regulates misfolded protein clearance in cancer cells and contributes to lysosome function and that reduction of PKR expression in cancer cells impairs lysosome function. Lysosomes have recently emerged as promising targets for cancer therapy [24–27]. Researchers have found that the characteristics of lysosomes, including their size, stability, intracellular localization, cathepsin expression, and enzymatic activity, differ in cancer cells and normal cells [24–28]. On the basis of these findings, we hypothesized that destabilizing PKR-associated lysosome function is an effective approach to cancer therapy. To test this hypothesis, we screened a 10,000-compound library, and identified PKR-associated compound 1 (Pac 1) that selectively induced cell death in cancer cells and inhibited the growth of human tumor xenografts in mice without toxicity. Our findings demonstrated that treatment with Pac 1 binds to PI4K2A and disrupts PI4K2A/PKR network, contributing directly to destabilization of cancer cell lysosomes and triggering cell death. PI4K2A is targeted to the *trans*-Golgi network and lysosomes [29–31]. Recently, an increasing number of studies have identified PI4K2A as a potential target for breast cancer therapy [32–34]. These results improved our understanding of PI4K2A/PKR lysosome networks may lead to the development of unique and highly specific targeted therapies for cancer.

## Results

### PKR regulates misfolded prion protein (PrP) clearance

**Fig. 1:**
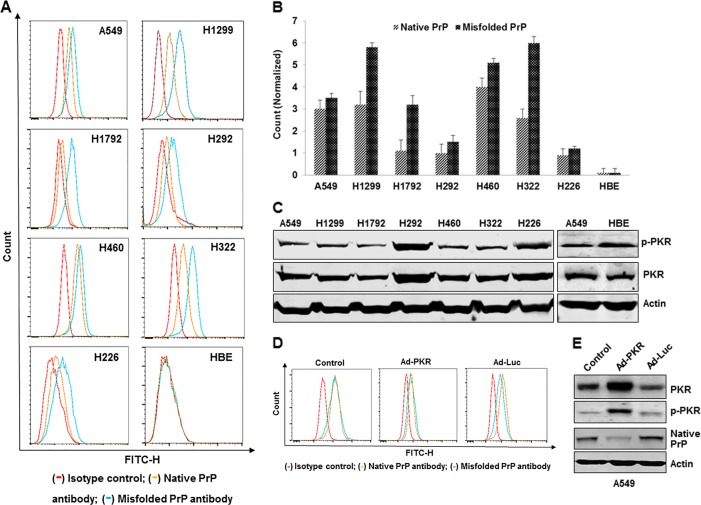
Correlation of PKR expression with misfolded PrP levels in lung cancer cells. **a** FACS analysis of native and misfolded PrPs in human lung cancer cell lines and normal HBE cells. **b** Average numbers of native and misfolded PrPs in human lung cancer cell lines and HBE cells as determined using FACS. Native and misfolded PrP values were normalized according to isotype control values. Experiments were performed in triplicate; data are presented as means. **c** Western blot of seven human lung cancer cell lines and one normal cell line (HBE) for expression of PKR and p-PKR protein. Actin was used as a loading control. **d** FACS analysis of native and misfolded PrPs in A549 cells 48 h after transfection with Ad-Luc or Ad-PKR (2500 viral particles/cell). **e** Western blot analysis of native PrPs in A549 cells 48 h after transfection with Ad-Luc or Ad-PKR (2500 viral particles/cell)

To investigate the role of PKR in clearance of misfolded proteins in cancer cells, we developed antibody specific for misfolded PrP. We examined the expression of native and misfolded PrP in cancer cells. Specifically, we measured misfolded PrP expression levels in seven human lung cancer cell lines and one normal human bronchial epithelial (HBE) cell line using fluorescence-activated cell sorting (FACS) with the AMF-1c-120 antibody. Native and misfolded PrPs were expressed in all cancer cell lines examined but not in normal HBE cells (Fig. 1a, b). Furthermore, misfolded PrPs were much more strongly expressed than were native PrPs in those cell lines. The two cancer cell lines with the highest phosphorylated PKR (p-PKR) expression levels (H292 and H226) had lower levels of native and misfolded PrP expression than did cell lines with lower p-PKR levels (Fig. 1a–c). In A549 cells, induction of PKR expression reduced levels of native as well as misfolded PrP expression (Fig. 1d). Western blotting results indicated that induction of PKR expression reduced levels of native PrP expression in A549 lung cancer cells (Fig. 1e).

**Fig. 2:**
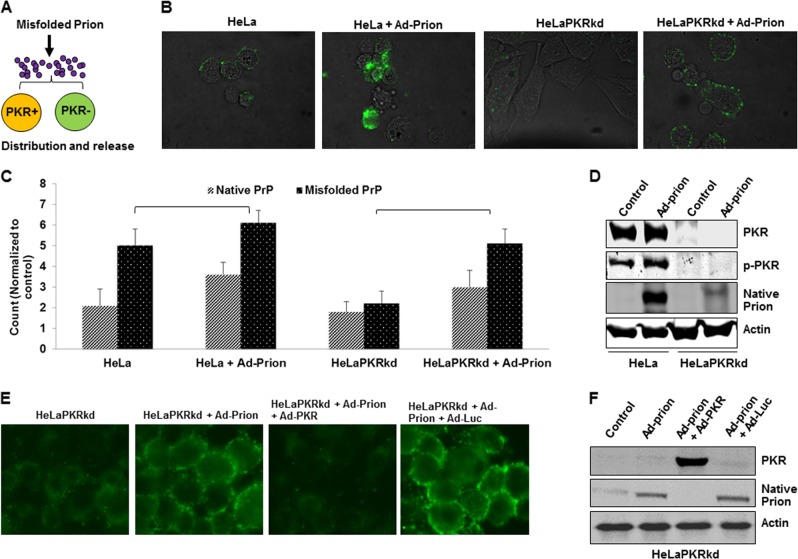
PKR affects the distribution and release of misfolded PrPs. **a** Our experimental model. **b** Immunofluorescent confocal microscopic analysis of native PrP expression in HeLa and HeLaPKRkd cells 48 h after transfection with Ad-Prion (2500 viral particles/cell) or without transfection. HeLaPKRkd cells exhibited excess surface PrPs (mostly in misfolded form) after Ad-Prion transfection. **c** Average levels of native and misfolded PrP expression in HeLa and HeLaPKRkd cells 48 h after transfection with Ad-Prion. Native and misfolded PrP values were normalized according to isotype control values. Experiments were performed in triplicate; data are presented as means. **d** Western blot of HeLa and HeLaPKRkd cells for expression of PKR, p-PKR, and PrPs 48 h after transfection with Ad-Prion (2500 viral particles/cell) or without transfection. Actin was used as a loading control. **e** Immunofluorescent confocal microscopic images of native PrP expression in HeLaPKRkd cells and HeLaPKRkd cells with re-expression of PKR (or Luc) 48 h after transfection with Ad-Prion (2500 viral particles/cell) or without transfection. **f** Western blot analysis of native PrPs in HeLaPKRkd cells with re-expression of PKR (or Luc) 48 h after transfection with Ad-Prion (2500 viral particles/cell) or without transfection

We next examined how expression of PKR in cancer cells regulates misfolded PrP distribution and release (Fig. 2a). We induced misfolded PrP expression in HeLa human cervical cancer cells and PKR-knockdown HeLa (HeLaPKRkd) cells by transfecting them with an adenoviral PrP vector (Ad-Prion). We then analyzed intracellular and surface induction of native PrP expression using confocal microscopy and FACS. Transfection with Ad-Prion resulted in abnormal local overexpression of PrP (possibly intracellular PrP accumulation in lysosomes) in HeLa cells and surface expression of PrP in HeLaPKRkd cells (Fig. 2b). Furthermore, Ad-Prion induced higher levels of surface misfolded PrP expression in HeLaPKRkd cells than in HeLa cells (Fig. 2c). We next evaluated expression of PKR, p-PKR, and intracellular PrPs in Ad-Prion-transfected HeLa and HeLaPKRkd cells using western blotting. Ad-Prion transfection caused accumulation of intracellular PrP and induction of PKR and p-PKR expression in HeLa cells but not HeLaPKRkd cells, in which the amount of intracellular PrP was reduced (Fig. 2d). We further induced PrP expression in PKR-deficient HeLaPKRkd cells as well as HeLaPKRkd cells re-expressing PKR. We analyzed intracellular and surface induction of native PrP expression using confocal microscopy. As expected, transfection with Ad-Prion resulted in surface expression of PrP in HeLaPKRkd cells (Fig. 2e). However, re-expression of PKR but not luciferase (Luc) in HeLaPKRkd cells reduced the surface expression of PrP. Western blotting results indicated that induction of PKR expression reduced levels of native PrP expression in Ad-Prion-transfected HeLaPKRkd cells (Fig. 2f). These data make it possible to hypothesize that induction of low levels of intracellular PrP expression in Ad-Prion-transfected PKR-deficient cells could be attributable to quick release of PrPs into the extracellular space via multivesicular bodies (MVBs)/exosomes. We also hypothesized that PKR plays a significant role in lysosome function, and promotes misfolded protein degradation, thereby reducing the release of these proteins in cancer cells. These hypotheses are according with the PKR involvement in diseases related to lysosomes break down abnormal proteins [13, 14].

### PKR regulates lysosomal degradation of misfolded PrPs

**Fig. 3:**
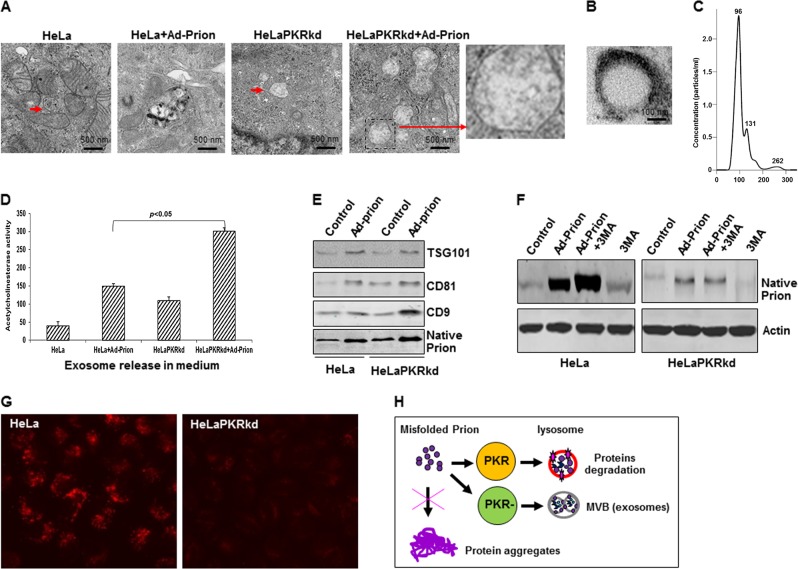
PKR is involved in lysosomal degradation of misfolded proteins. **a** TEM analysis of HeLa and HeLaPKRkd cells 48 h after transfection with Ad-Prion or without transfection. The scale bars represent 500 nm. **b** The vesicular structure of exosomes as shown by TEM. The scale bar represents 100 nm. **c** Average exosome size was determined by NanoSight. **d** Average exosome counts in HeLa and HeLaPKRkd cell media 48 h after transfection with Ad-Prion. Experiments were performed in triplicate; data are presented as means. **e** Western blot of expression of TSG101, CD81, and native PrP in exosomes in the HeLa and HeLaPKRkd cell media 48 h after transfection with or without Ad-Prion. **f** Western blot of HeLa and HeLaPKRkd cells for expression of PrP 48 h after transfection with Ad-Prion (2500 viral particles/cell) and treatment with 3MA (1 mM). Actin was used as a loading control. **g** Microscopic images of HeLa and HeLaPKRkd cells subjected to LysoTracker Red staining. **h** Proposed mechanisms for prevention of misfolded proteins by PKR-mediated lysosome function in cancer cells

To determine whether PKR dependent lysosome function promotes misfolded protein degradation, we further examined lysosomes in cells after induction of misfolded PrP expression using transmission electron microscopy (TEM). We observed that HeLa cells but not HeLaPKRkd cells had lysosomes and that the latter cells had MVBs/exosomes (Fig. 3a). Ad-Prion transfection induced destruction of lysosomes in HeLa cells and increased the number of MVBs/exosomes and spurred formation of dilated MVBs in HeLaPKRkd cells. The vesicular structure of exosomes isolated from the supernatant of Ad-Prion-transfected HeLaPKRkd cells was analyzed using TEM (Fig. 3b), and average exosome size was determined using NanoSight (Fig. 3c). Because exosomes are rich in proteins such as acetylcholinesterase, we analyzed the exosomal fraction by measuring the activity of acetylcholinesterase. We found higher acetylcholinesterase activity, indicating a larger quantity of released exosomes, in the supernatant of Ad-Prion-transfected HeLaPKRkd cells than in that of Ad-Prion-transfected HeLa cells (Fig. 3d). In addition, we detected higher amounts of PrPs and exosome markers (TSG101, CD81, and CD9) in exosomes from the supernatant of Ad-Prion-transfected HeLaPKRkd cells than in that of Ad-Prion-transfected HeLa cells (Fig. 3e). We also found that PKR wild-type and PKR-deficient cancer cells responded to the presence of misfolded PrPs differently. We next investigated the involvement of lysosomes in PrP degradation. We transfected HeLa and HeLaPKRkd cells with Ad-Prion for 48 h in the presence or absence of the lysosome inhibitor 3-methyladenine (3MA; 1 mM). 3MA prevented prion degradation in HeLa cells but not in HeLaPKRkd cells (Fig. 3f). To determine whether PKR contributes directly to lysosome function, we evaluated this function in PKR wild-type (HeLa) and stable PKR-knockdown (HeLaPKRkd) cells using the acidophilic dye LysoTracker Red (Life Technologies). HeLaPKRkd cells displayed markedly lower numbers of acidic vesicular organelles than did HeLa cells (Fig. 3g), suggesting the presence of a lysosomal defect or abnormality in HeLaPKRkd cells. These results demonstrated that PKR contributes to lysosome function and promotes misfolded protein degradation, thereby preventing the release of these proteins in cancer cells. We therefore proposed that (1) PKR-positive cancer cells respond to the presence of abnormal proteins such as misfolded proteins by promoting lysosome function, thereby avoiding the cell death that results from aggregation of these harmful proteins (Fig. 3h); and (2) cancer cells may reduce or deplete PKR and increase the number of MVBs/exosomes to release misfolded proteins. These data demonstrated that PKR is involved in lysosomal degradation of misfolded proteins and that PKR may contribute directly to lysosome function.

### Anticancer effects of PKR-targeting compounds depend on PKR expression status

**Fig. 4:**
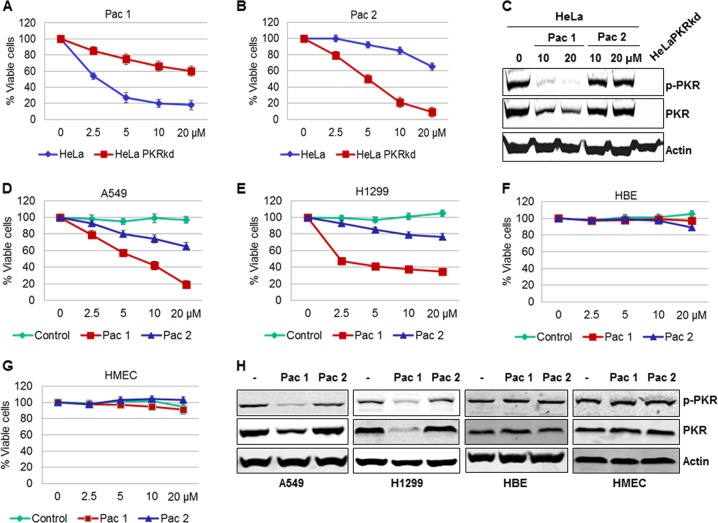
Treatment with PKR-modulating compounds inhibits cancer cell growth in vivo. **a**, **b** Viability of HeLa and HeLaPKRkd cells as determined using an SRB assay 72 h after treatment with Pac 1 (**a**) or Pac 2 (**b**). Experiments were performed in triplicate, and the data are presented as means. **c** Representative western blot of expression of PKR and p-PKR protein in HeLa cells 72 h after treatment with Pac 1 or Pac 2. HeLaPKRkd cells were used as controls. **d–g** Viability of lung cancer cells (**d**, **e**) and normal cells (**f**, **g**) 72 h after treatment with Pac 1 or Pac 2 as determined using an SRB assay. HMEC human mammary epithelial cells. **h** Western blot for expression of PKR and p-PKR protein in lung cancer and normal cells 72 h after treatment with 5 μM Pac 1 or Pac 2

**Fig. 5:**
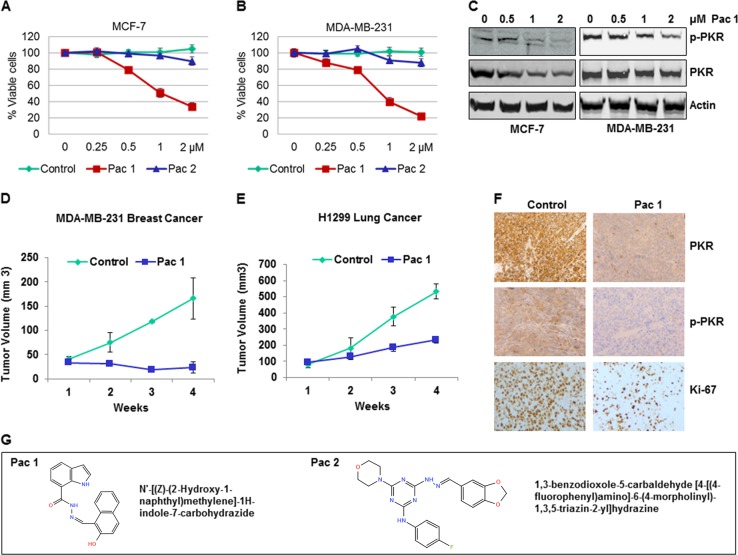
Tumor xenografts in an orthotopic mouse model treated by Pac 1. **a**, **b** Viability of two breast cancer cell lines as determined using an SRB assay 72 h after treatment with Pac 1 or Pac 2. **c** Western blot of the expression of PKR and p-PKR protein in the two breast cancer cell lines 72 h after treatment with Pac 1 at various doses. **d**, **e** Growth of MDA-MB-231 breast (**d**) and H1299 lung (**e**) tumor xenografts in an orthotopic mouse model. Tumor growth in both models was markedly lower in tumors intravenously injected with Pac 1 than in untreated control tumors. Tumor volumes (*y*-axes) were measured in five to six mice per group over 28 days. The data are presented as means (error bars, standard deviation). **f** Immunohistochemical analysis of H1299 lung tumors showing that the growth inhibition observed in Pac 1-treated mice was caused by reductions in PKR, p-PKR, and Ki-67 protein expression. **g** The chemical structures of Pac 1 and Pac 2

Because PKR wild-type HeLa cells have lysosomes and PKR-deficient HeLaPKRkd cells have MVBs/exosomes, we hypothesized that using these two cell lines would enable us to identify compounds selectively targeting rich lysosomes in PKR-positive tumors or rich MVBs/exosomes in PKR-negative tumors. To identify compounds that can target lysosomes in PKR-positive cells or MVBs/exosomes in PKR-negative cells, we screened the 10,000-compound ChemBridge library for compounds with different effects on the growth of HeLa and HeLaPKRkd cells. By examining morphologic changes in the cells using microscopy and cell growth inhibition using a sulforhodamine B (SRB) assay, we identified Pac 1 and Pac 2. Microscopic analysis of morphologic changes in HeLa and HeLaPKRkd cells demonstrated that treatment with Pac 1 suppressed the growth of HeLa cells more effectively than did treatment with Pac 2, whereas Pac 2 suppressed the growth of HeLaPKRkd cells more effectively than did Pac 1 (data not shown). We next used SRB assays to determine cell viability after treatment with Pac 1 or Pac 2. The viability of HeLa cells was suppressed more effectively by Pac 1 than by Pac 2, whereas the viability of HeLaPKRkd cells was suppressed more effectively by Pac 2 (Fig. 4a, b). These results suggested that sensitivity to Pac 1 and Pac 2 depends on PKR status, as PKR-positive cells were sensitive to Pac 1, whereas PKR-negative cells were sensitive to Pac 2. We next examined the effects of Pac 1 and Pac 2 on PKR protein expression in HeLa cells using western blot analysis. We found that treatment with Pac 1 but not Pac 2 significantly reduced the expression of PKR and p-PKR (Fig. 4c). In addition, two tested human lung cancer cell lines (A549 and H1299) were more sensitive to treatment with Pac 1 than that with Pac 2 (Fig. 4d, e). We observed no reduction in normal cell viability after treatment with Pac 1 or Pac 2 (Fig. 4f, g). Using western blotting, we confirmed that Pac 1 but not Pac 2 markedly reduced PKR and p-PKR expression in A549 and H1299 cells but not in normal HBE or human mammary epithelial cells (Fig. 4h). In addition, treatment with Pac 1 inhibited the growth of two breast cancer cell lines (MCF-7 and MDA-MB-231) (Fig. 5a, b) and does dependently reduced PKR expression (Fig. 5c). Because most of the cancer cell lines we tested were more sensitive to Pac 1 than to Pac 2, we further investigated Pac 1. Intravenous injection of mice with Pac 1 inhibited the growth of MDA-MB-231 (Fig. 5d) and H1299 (Fig. 5e) tumor xenografts over 4 weeks without any toxicity (Supplementary material). Pac 1 also dramatically suppressed the expression of PKR, p-PKR, and Ki-67 protein in H1299 tumors (Fig. 5f). Figure 5g shows the structures of Pac 1 and Pac 2. Taken together, these data demonstrated that treatment with Pac 1 did not affect normal cells but selectively killed cancer cells depending on their PKR status.

### PKR/PI4K2A lysosome network is a potential target for Pac 1, and are associated with poor prognosis in breast cancer patients

**Fig. 6:**
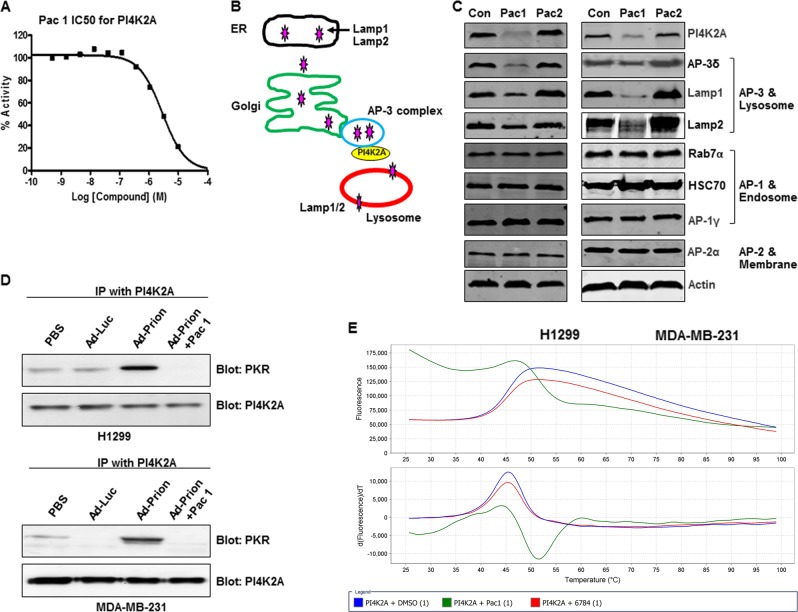
Treatment with Pac 1 inhibits PI4K2A expression and impairs lysosome function in cancer cells. **a** The half-maximal inhibitory concentration (IC50) of Pac 1 for PI4K2A kinase as determined by Reaction Biology Corp. using a high-throughput enzymatic assay. **b** Mechanism of action of PI4K2A on the AP-3 complex in cancer cells. ER endoplasmic reticulum. **c** Western blot of expression of AP-1, AP-2, and AP-3 complex-associated proteins in lung and breast cancer cells 72 h after treatment with 5 μM Pac 1 or Pac 2. Actin expression was used as a loading control (Con). **d** Protein fractions obtained from H1299 and MDA-MB-231 cells treated with PBS, Ad-Luc, Ad-Prion (2500 viral particles/cell), or Ad-Prion plus Pac 1 (5 μM) for 48 h were immunoprecipitated with antihuman PI4K2A and assayed for PKR or PI4K2A protein. **e** Differential scanning fluorimetry curve showing that Pac 1 bound to PI4K2A and caused a decrease in the melting temperature of PI4K2A protein

To determine the mechanism of cell growth inhibition by Pac 1, we performed a high-throughput enzymatic screening assay to identify targets of Pac 1. We screened PKR as well as a recombinant human protein panel consisting of 366 wild-type kinases, 175 mutant kinases, 20 atypical kinases, and 17 lipid kinases. We found that Pac 1 did not inhibit PKR kinase activity. However, we did observe that Pac 1 inhibited the activity of PI4K2A kinase (Fig. 6a). Pac 1 exhibited the greatest inhibitory effect on PI4K2A kinase activity (half-maximal inhibitory concentration, 2.5 μmol/L). PI4K2A is associated with adapter-related protein complex 3 (AP-3) protein and is involved in the biogenesis of lysosomes by directing the sorting of lysosomal membrane proteins to lysosomes (Fig. 6b) [29, 30].

We next used western blotting to evaluate proteins known to be involved in AP-1/2/3 complexes in lung (H1299) and breast (MDA-MB-231) cancer cells after treatment with Pac 1 or Pac 2. Unlike control and Pac 2-based treatment, Pac 1 (5 μM) significantly inhibited PI4K2A, Lamp1, Lamp2, and AP-3δ expression in H1299 and MDA-MB-231 cells (Fig. 6c). We did not observe reduction of expression of proteins associated with AP-1 (Rab7α, HSC70, and AP-1γ) or AP-2 (AP-2α) in either cancer cell line 72 h after treatment with Pac 1 or Pac 2. AP-1 and AP-2 mediate protein sorting in the endosome, *trans*-Golgi network, and plasma membrane. We found that inhibition of PI4K2A expression decreased the stability of AP-3 and associated client proteins.

Next, we investigated whether Pac 1 disrupts the PI4K2A-associated PKR network and contributes directly to destabilization of cancer cell lysosomes. We immunoprecipitated H1299 and MDA-MB-321 cells treated with phosphate-buffered saline (PBS), Ad-Luc alone, Ad-Prion alone, or a combination of Ad-Prion and Pac 1 with an anti-PI4K2A antibody and then immunoblotted them with an anti-PKR antibody. Figure 6d shows that the amount of PKR coimmunoprecipitated with PI4K2A was dramatically greater in both cancer cell lines after treatment with Ad-Prion. However, the amount of PKR coimmunoprecipitated with PI4K2A decreased to undetectable levels in both cell lines after treatment with the combination of Ad-Prion and Pac 1. Pac 1 produced a substantial change in the differential scanning fluorimetry curve as compared with the PI4K2A protein alone (Fig. 6e). We detected that Pac 1 binds to PI4K2A and causes a decrease in the melting temperature of PI4K2A protein. A differential scanning fluorimetry assay demonstrated that Pac 1 not only interacted directly with PI4K2A but also destabilized it. Taken together, these results suggested that treatment with Pac 1 inhibits cancer cell growth via impairment of lysosome function triggered by disruption of the PKR/PI4K2A lysosome network.

**Fig. 7:**
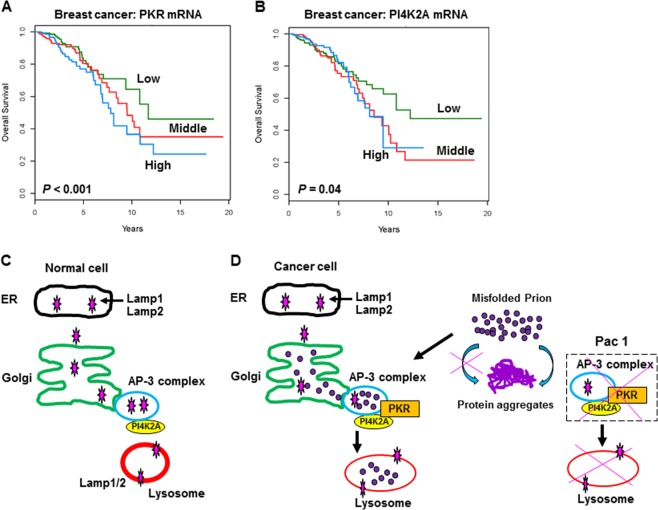
Prognostic significance of PKR and PI4K2A expression in cancer patients. **a**, **b** PKR and PI4K2A are encoded by the EIF2AK2 and PI4K2A genes, respectively. In the TCGA data analysis, high levels of EIF2AK2 (**a**) and PI4K2A (**b**) gene expression were associated with low overall survival rates in breast cancer patients. **c** Our proposed mechanism of action of PI4K2A network in normal cells. ER, endoplasmic reticulum. **d** Our proposed mechanism of action of Pac 1 in cancer cells

In an analysis of data in The Cancer Genome Atlas (TCGA) (RNA sequencing data set; https://tcga-data.nci.nih.gov/tcga/). We evaluated the association between PKR (encoded as EIF2AK2) or PI4K2A gene expression and overall survival in breast cancer patients. We found that high levels of EIF2AK2 and PI4K2A gene expression were associated with low overall survival rates in breast cancer patients (Fig. 7a, b).

## Discussion

In this study, we discovered a new function of PKR in cancer cells in that it regulates misfolded protein clearance and contributes to lysosome function. Regulation of misfolded proteins clearance in cancer is unknown. Authors have suggested that cancer cells produce larger quantities of misfolded proteins than normal cells do [9–12, 17, 35, 36]. The lysosomes in the cytoplasm collect the unfolded/misfolded proteins that the cell does not use and break them down, which is an important host defense/clearance mechanism [3–5, 24, 25]. Also, the role of PKR in preventing the spread of viruses has been well documented [14, 18, 21, 23, 37]. PKR can respond to various stress signals, such as pathogens, nutrient depletion, cytokines, irradiation, and endoplasmic reticulum stress [18, 37–40]. However, because of a lack of anti-misfolded protein antibodies, investigation of these proteins is difficult. Only one misfolded anti-PrP antibody was available to us for use in our experiments. We found that misfolded PrPs were expressed in cancer cells but not normal cells. We also observed that induction of PKR expression reduced misfolded PrP expression in lung cancer cells. After transfection of PKR wild-type and PKR-deficient cells with Ad-Prion, PKR wild-type cells produced high intracellular, low surface, and low extracellular levels of PrP, whereas PKR-deficient cells produced low intracellular, high surface, and high extracellular PrP levels. These data demonstrated that PKR is involved in lysosomal degradation of misfolded proteins.

Our results also demonstrated that PKR contributes directly to lysosome function and that loss of PKR in cancer cells impairs lysosome function and reduces cell viability. Lysosomes play an important role in protein recycling and energy metabolism in cancer cells [41]. Studies have demonstrated that rapidly dividing cells such as cancer cells need effective lysosomal function for proliferation. Several changes in lysosomes occur during oncogenic transformation, including changes in their volume, stability, composition, and subcellular localization [24–26, 42, 43]. Authors reported that leukemia cells had markedly larger lysosomes than did normal cells and overexpressed lysosomal biogenesis genes [44]. In addition, cancer cells depend on lysosome function for survival, as they help the cells adapt to nutrient stress and eliminate abnormal proteins [1, 2]. We propose that the PI4K2A/AP-3 network regulates lysosome function in normal cells (Fig. 7c) and that a PKR-associated PI4K2A/AP-3 network responds to accumulated misfolded proteins by promoting lysosome function in cancer cells, thereby preventing the cell death that results from aggregation of these harmful proteins (Fig. 7d). We further propose that some cancer cells may respond to misfolded proteins by reducing the expression of PKR and increasing the number of MVBs/exosomes to remove these proteins. Further studies are needed to test this.

Normal cells do not produce misfolded proteins. However, rapidly dividing, invasive cancer cells are strongly dependent on lysosomal function to remove misfolded proteins and maintain organelle homeostasis. Therefore, therapeutically targeting the misfolded protein-associated lysosome network will result in accumulation of misfolded proteins and aggregates of these proteins and cause cancer cell death. Investigators have proposed several strategies to target lysosomes for cancer treatment, including use of agents that permeabilize lysosome membranes and cathepsin inhibitors [27]. In the present study, we screened a compound library and identified two compounds that target PKR-mediated lysosome function: Pac 1 and Pac 2. Treatment with them did not affect the viability of normal cells, and cancer cells’ sensitivity to these compounds depended on their PKR statuses. Our findings reported herein demonstrated that Pac 1 directly inhibits the expression and activity of PI4K2A kinase but not other kinases. PI4K2A is targeted to the *trans*-Golgi network and lysosomes [29–31]. Recently, an increasing number of studies have identified PI4K2A as a potential target for breast cancer therapy [32–34]. We found that Pac 1 not only directly inhibited PI4K2A expression but also destabilized AP-3 and associated client proteins, such as Lamp1/2 and PKR. Our findings demonstrated that Pac 1 disrupts the PI4K2A/PKR network, contributing directly to destabilization of cancer cell lysosomes and triggering cell death (Fig. 7d). In an analysis of data in TCGA, we found that high levels of EIF2AK2 and PI4K2A gene expression were associated with low overall survival rates in breast cancer patients. These results demonstrate that targeting of a PI4K2A/PKR lysosome complex may be an effective approach for cancer therapy. Further studies are needed to determine the precise mechanism by which PKR interacts with lysosomes and confirm that Pac 1 targets the PI4K2A/PKR network in misfolded proteins that accumulate in cancer cells.

In conclusion, the results of the present study demonstrated that human lung cancer cells but not normal cells release large quantities of misfolded PrPs and that PKR prevents this release. We found that PKR contributes directly to lysosome function and that loss of PKR in cancer cells impairs lysosome function. Treatment with Pac 1 disrupts the PI4K2A/PKR network, impairing lysosome function and inhibiting growth of various types of cancer cells in vitro and in vivo. On the basis of these findings, we conclude that using Pac 1 to target PI4K2A/PKR lysosome networks is a promising approach to cancer therapy.

## Materials and methods

### Cell lines

Human lung cancer (A549, H1299, H1792, H460, H322, H292, and H226), breast cancer (MDA-MB-231 and MCF-7), and colon cancer (LOVO) cell lines were obtained from the ATCC (Manassas, VA). All cell lines were maintained in Dulbecco’s modified essential medium or RPMI 1640 medium supplemented with 10% fetal bovine serum, 10 mM glutamine, 100 U/mL penicillin, and 100 mg/mL streptomycin (Life Technologies, Inc., Grand Island, NY). The normal HBE cell line was purchased from Clonetics (Walkersville, MD). HBE cells were cultured in serum-free keratinocyte medium (Invitrogen, Carlsbad, CA). Normal human mammary epithelial cells were obtained from the ATCC. HeLa (PKR wild-type) and HeLaPKRkd cells were provided by CS (University of California, Santa Barbara) and were described previously [23, 45].

### Antibodies and chemicals

An antibody against PKR (sc-707) was obtained from Santa Cruz Biotechnology (Santa Cruz, CA). Anti-p-PKR (pT451, 1120-1) and -p-eIF2α (S51) antibodies were obtained from Epitomics (Burlingame, CA), and an anti-paxillin antibody (catalog #2542) was obtained from Cell Signaling Technology (Beverly, MA). A mouse anti-β-actin antibody was obtained from Sigma-Aldrich (St. Louis, MO). The misfolded anti-PrP antibody AMF-1c-120 was provided by Amorfix Life Sciences Ltd (Mississauga, ON, Canada). Rabbit monoclonal antibodies against native PrP (EP1802Y) and BAG3 (catalog #ab92309) were purchased from Abcam (Cambridge, UK). Alexa Fluor 488 goat anti-rabbit immunoglobulin G (H + L) was purchased from Life Technologies.

### Adenoviral transfection and western blotting

An adenoviral vector carrying the wild-type PKR gene (Ad-PKR) previously developed by our group was used in this study. Cells were transfected with Ad-PKR or Ad-Luc, and cell extracts were prepared 48 h later. The cell extracts were subjected to western blotting [46]. To determine the roles of lysosomes in PKR-mediated protein degradation, lysosome function was inhibited using 3MA (Sigma-Aldrich). Cancer cells were seeded in six-well plates (2 × 10^5^ cells/well) in RPMI 1640 medium overnight. The next day, Ad-Prion or control Ad-Luc was added for 24 h, and 3MA (1 mM) was added for an additional 24 h. Cells were then collected for western blotting.

### LysoTracker red staining, confocal microscopy, and tem

The acidophilic dye LysoTracker Red stains lysosomes. Cancer cells (1 × 10^5^ cells/well) were grown on two-well chamber slides (Becton Dickinson Labware, Bedford, MA) to 70% confluence and then treated with PKR siRNA or control siRNA. Forty-eight hours later (when cells had grown to 70–80% confluence), the medium was replaced with medium containing 75 nM LysoTracker Red. The cells were incubated with LysoTracker Red for 1 h and then rinsed one time with 1 mL of PBS. The cells were kept in PBS for microscopic imaging.

For confocal microscopic imaging of Lamp1, HeLa, and HeLaPKRkd cells (1 × 10^5^ cells/well) were grown on two-well chamber slides to 70% confluence and then transfected with Ad-Lamp1-GFP (Life Technologies). The cells were washed with PBS after 48 h and fixed with 4% paraformaldehyde/PBS for imaging.

For confocal microscopic imaging of PrP, HeLa, and HeLaPKRkd cells (2–4 × 10^4^ cells/well) were grown on eight-well chamber slides (Falcon, Big Flats, NY) to 70% confluence and then transfected with Ad-Prion. The cells were washed with PBS after 48 h and fixed with 4% paraformaldehyde/PBS for confocal microscopy as described previously [47]. Cells were blocked with 1% normal goat serum for 1 h and then incubated for 1 h at a dilution of 1:100 with the primary native anti-PrP antibody. Next, the slides were washed to remove the primary antibody, rinsed with PBS, and exposed to the secondary antibody, a rabbit antibody-Alexa Fluor 488 conjugate (Molecular Probes, Life Technologies), for about 30 min at room temperature. The slides were then mounted with ProLong Gold antifade reagent containing 4′,6-diamidino-2-phenylindole (Invitrogen) and analyzed under a FluoView FV500 laser confocal microscope (Olympus America, Melville, NY) after adjustment for background staining.

TEM analyses were performed in our core facility.

### Flow cytometry

For detection of misfolded proteins, primary normal cells, primary tumor cells, or cancer cells transfected with adenoviral vectors (Ad-PKR, Ad-Luc, or Ad-Prion) were dissociated with cell dissociation buffer (Life Technologies; catalog #13151014). The collected cells were washed with PBS and suspended in FACS staining buffer (2% fetal bovine serum and 0.02% azide in PBS), and the misfolded anti-PrP antibody AMF-1c-12 (20 μL) or native anti-PrP antibody EP1802Y (20 μL) was added. The cells then were incubated at 4 °C for 30 min and spun at a relative centrifugal force of 500 for 5 min at 4 °C. They then were washed three times with 100 μL of FACS staining buffer. After the final wash, the cells were resuspended in a secondary antibody solution (50 μL of 3 μg/mL anti-rabbit IgG AF488), incubated at 4 °C for 30 min, and spun at a relative centrifugal force of 500 for 5 min at 4 °C. Next, they were washed three times with 100 μL of FACS staining buffer. After the final wash, the cells were resuspended in 100 μL of a propidium iodide solution (1 μg/mL) and subjected to FACS analysis.

### Isolation and quantitation of exosomes

Exosomes were collected from 5 mL of media from cell culture (1–2 × 10^7^). The culture media were collected, subjected to centrifugation at 800 × *g* for 10 min to sediment the cells, and centrifuged at 12,000 × *g* for 30 min to remove the cellular debris. The exosomes were separated from the supernatant via centrifugation at 100,000 × *g* for 2 h. The exosome pellet was washed once in a large volume of PBS and resuspended in 100 μL of PBS to yield the exosome fraction. The amount of released exosomes was quantified by measuring the activity of acetylcholinesterase, an enzyme that is specifically directed to these vesicles. Acetylcholinesterase activity was assayed by following a procedure described previously [48]. Briefly, 25 μL of the exosome fraction was suspended in 100 μL of phosphate buffer and incubated with 1.25 mM acetylthiocholine and 0.1 mM 5,5′-dithiobis(2-nitrobenzoic acid) in a final volume of 1 mL. The incubation was carried out in cuvettes at 37 °C, and the change in absorbance at 412 nm was observed continuously. The data reported represent the enzymatic activity after 20 min of incubation.

### Analysis of in vivo tumor growth after treatment with Pac 1

For in vivo tumor studies, MDA-MB-231 or H1299 cells (~1 × 10^6^) were resuspended in 0.1 mL of PBS and injected subcutaneously into the flanks of female severe combined immunodeficiency mice. When the resulting tumors reached 100–150 mm^3^ in volume, the mice were stratified into groups of eight animals, with each group having approximately equal mean tumor volumes, and administered intravenous injection of Pac 1. The animals were weighed weekly, and their tumor diameters were measured twice weekly. When a tumor reached 2000 mm^3^ or became necrotic, the animal was killed. Tumors obtained from mice that did or did not receive Pac 1 were analyzed immunohistochemically for PKR, p-PKR, and Ki-67 protein expression.

### Thermal shift assay

Recombinant PI4K2A protein purified from a plasmid encoding PI4K2A_76-465_ protein was provided by Boura [49]. A thermal shift assay was performed using a 7500 Fast Real-Time PCR System (Applied Biosystems). Each reaction solution contained 5 mmol/L PI4K2A, 5 SYPRO Orange Protein Gel Stain (Sigma-Aldrich), and the test compounds in 20 mL of buffer (50 mmol/L HEPES, pH 7.5, 150 mmol/L NaCl, 2 mmol/L MgCl_2_), which was heated from 25 to 95 °C at a 1% ramp rate. The melting temperature was calculated using the Boltzmann fitting method with the Protein Thermal Shift software program (version 1.1; Applied Biosystems). Each reaction was repeated three times.

### Cell viability assays, toxicity study, immunoprecipitation analysis and kinases activity assay

The method and materials for these assays are in Supplementary information.

### Statistical analysis

In vitro data reported in the figures represent the means (±standard deviation) from three independent experiments. In evaluating differences between treated and untreated groups. The differences between treatment groups in xenograft experiments were determined by using a one-sided exact Wilcoxon–Mann–Whitney test. *A P* value less than 0.05 was considered significant.

## Supplementary information

Supplemental Material
