# Supplementary material for: Therapeutic targeting of the PI4K2A/PKR lysosome network is critical for misfolded protein clearance and survival in cancer cells
Source: Oncogene. 2019 Sep 25;39(4):801–13. doi: 10.1038/s41388-019-1010-4 (PMC6976521; doi:10.1038/s41388-019-1010-4)
Supplement: Supplementary file 1 — Supplemental Material [file 41388_2019_1010_MOESM1_ESM.docx]

**Therapeutic Targeting of the PI4K2A/PKR Lysosome Network is Critical for Misfolded Protein Clearance and Survival in Cancer Cells** Apar Pataer et al

**Supplementary information**

**Methods**

**Chemical library**

A chemical library of 10,000 compounds and their analogs was obtained from ChemBridge Corporation. The chemicals in this library were provided at a concentration of 5 mg/mL in dimethyl sulfoxide. Each compound was dissolved in dimethyl sulfoxide to a concentration of 10 mM and stored at 4°C as a master stock solution.

**Cell viability assays**

For SRB assays, cells (2-8 × 10^3^/well in 100 μL of culture medium) were seeded in 96-well flat-bottomed plates and treated the next day with compounds at the indicated concentrations. After the indicated treatments, cells were fixed with trichloroacetic acid. The protein was stained with SRB, and the optical density at 570 nm was determined. Relative cell viability was calculated by setting the viability rate of the control cells (exposed only to dimethyl sulfoxide) at 100% and comparing the viability of the treated cells with that of the controls. The experiments were performed at least three times for each cell line. The viability of leukemia cell lines was determined by using a 3-(4,5-[di](http://en.wikipedia.org/wiki/Di-)[methyl](http://en.wikipedia.org/wiki/Methyl)[thiazol](http://en.wikipedia.org/wiki/Thiazole)-2-yl)-2,5-di[phenyl](http://en.wikipedia.org/wiki/Phenyl)tetrazolium bromide assay.

**Identification of PKR-associated compounds and cytotoxicity studies**

The library of 10,000 compounds was screened for those with different effects on the growth of HeLa and HeLaPKRkd cells. For this screening, both HeLa and HeLaPKRkd cells were seeded in 96-well plates in parallel and treated with each compound at a final concentration of 5 μg/mL. Cells treated with dimethyl sulfoxide (final concentration, 1%) were used as controls. Treated cells were observed under a microscope to detect morphologic changes, and their viability was determined using SRB assays 2 and 3 days after treatment. The compounds initially observed to have different effects on HeLa and HeLaPKRkd cells were subjected to two confirmatory screenings.

For the SRB assay, cells (2-8 × 10^3^/well in 100 μL of culture medium) were seeded in 96-well flat-bottomed plates and treated the next day with compounds at the indicated concentrations. After treatment for the indicated times, cells were fixed with trichloroacetic acid. The cells were stained using SRB, and the optical density at 570 nm was determined. Relative cell viability was determined by setting the viability of the control cells (exposed only to dimethyl sulfoxide) at 100% and comparing their viability with that of the treated cells. The experiments were performed at least three times for each cell line.

**Anticancer effects of PKR-modulating compounds in *vivo***

To determine the toxicity of these compounds in mice, 50 mg/kg Pac 1 or Pac 2 was injected intravenously into six 10-week-old BALB/c mice (three mice per treatment) daily for 7 days. No weight loss or apparent adverse or distressing effects on the well-being of the mice were observed. Pathologists at MD Anderson carried out blood chemistry analysis and histopathologic examination of organs in the Pac 1- and Pac 2-injected mice. Blood analysis suggested that the mice receiving either compound had normal aspartate aminotransferase, serum glutamic-oxaloacetic transaminase, serum glutamic-pyruvic transaminase, alkaline phosphatase, creatine kinase, and lactate dehydrogenase levels. Histopathologic examination indicated no significant changes in any major organs, including the heart, lungs, liver, kidneys, pancreas, and spleen.

**Immunoprecipitation analysis**

Cancer cells were treated with PBS, Ad-Luc, Ad-Prion, Ad-Prion plus Pac 1 for 48 h, and then subjected to lysis in RIPA buffer (1 x PBS, 1% Nonidet P-40, 0.5% sodium deoxycholate, 0.1% sodium doclecyl sulfate (SDS). Cell lysates (500 µl [500 µg]) were incubated with primary antibody overnight at 4°C. Protein A/G agarose was added to the mix which was incubated for a further 4 h. Beads were pelleted by centrifugation at 2500 rpm for 5 min at 4°C and washed 4 times with 1 ml of RIPA buffer. After the last wash, 50 µl of 1X SDS-polyacrylamide gel electrophoresis sample buffer was added to the beads. This preparation was subjected to vortexing and then boiled for 5 min. It was centrifuged at 2500 rpm for 1 min before the supernatants were loaded on gels.

**Kinases activity assay**

The ADP-Glo kinase assay was used to evaluate the kinase activities of PKR, PI4K2A, PI4KIIb, PI4KIIIa, PI4KIIIb, PI3Ka, PI3Kb, PI3Kg by Reaction Biology.
